# Learner emotions in AI-assisted English as a second/foreign language learning: a systematic review of empirical studies

**DOI:** 10.3389/fpsyg.2025.1652806

**Published:** 2025-10-02

**Authors:** Songcun Zhang, Xing Liu

**Affiliations:** ^1^School of Foreign Languages, Ningbo University of Technology, Ningbo, Zhejiang, China; ^2^College of Languages and Cultures, North Minzu University, Yinchuan, Ningxia, China

**Keywords:** learner emotions, AI-assisted language learning, systematic review, empirical studies, enjoyment, anxiety, boredom, emotion regulation

## Abstract

**Introduction:**

The use of generative AI tools in language learning has attracted increasing academic attention. However, previous reviews rarely focus on learner emotions in AI-assisted English as a Second/Foreign Language (ESL/EFL) learning, a crucial aspect for second language (L2) development. The present study addresses this gap by examining 37 relevant empirical studies, including four pieces of grey literature, focusing on the characteristics of recent research on learner emotions in AI-assisted ESL/EFL learning, the emotional variables, and the influence of AI tools on students’ emotions.

**Methods:**

The researchers conducted a database search in Scopus, Web of Science, OpenGrey, and Google Scholar for relevant articles, following the PRISMA statement. Finally, 37 journal articles were selected in this study.

**Results:**

A review of findings indicates that AI tools have been widely utilized in more than 10 non-English-speaking countries, encompassing various English language skills, which suggests that AI-assisted ESL/EFL learning has gained popularity in L2 learning. Both positive and negative emotions were researched, including enjoyment, anxiety, boredom, interest, and shyness, as well as the relevant emotion regulation. Additionally, AI-driven assessment is a rapidly growing research trend. AI-assisted ESL/EFL learning can trigger students’ positive emotions and improve their English skills.

**Discussion:**

The results of this study provide a deeper understanding of the application of AI tools in ESL/EFL learning and their impact on the emotions of L2 learners in recent years. It will be useful for educators and researchers seeking to understand and evaluate AI-assisted ESL/EFL learning, as well as learners interested in using AI tools.

## 1 Introduction

Research on the emotional dimensions in the context of English language learning and teaching is booming. Previous studies have found that English language learners can experience both positive and negative emotions simultaneously (Dewaele et al., 2019; Dewaele and Li, 2020). MacIntyre and Vincze (2017) identified 19 fundamental emotions that are closely related to L2 learning motivation, based on their research in a German-as-a-foreign-language context of Italian secondary schools, with 10 positive emotions: joy, gratitude, serenity, interest, hope, pride, amusement, inspiration, awe and love, and 9 negative emotions: anger, contempt, disgust, embarrassment, guilt, hate, sadness, feeling scared and being stressed (Dewaele and Li, 2020; MacIntyre and Vincze, 2017). Actually, there are more variables in the applications of emotions in L2 research and practice. The most frequently researched emotions in L2 learning were enjoyment and anxiety (Dewaele and Li, 2020), followed by other variables such as happiness, passion, and satisfaction (Wang et al., 2021).

Understanding learner emotions is crucial for providing affective interventions and support to enhance learner motivation and self-concept (Hwang et al., 2020). Some intelligent emotion detection technologies are used to identify useful information from brain signals, enabling the understanding of learners’ feelings, such as happiness, sadness, fear, and calm (Chen et al., 2022). Accordingly, the connection between emotions and AI technologies is becoming closer and increasingly important. Some previous studies considered that AI technology can trigger EFL learners’ positive emotions and inhibit negative emotions, such as increasing their interest in language learning and decreasing language learning anxiety (Dizon, 2020; Wu and Li, 2024).

In recent years, the integration of artificial intelligence (AI) tools in English as a Second/Foreign Language (ESL/EFL) learning has offered a new and promising perspective for ESL/EFL learners to enhance their language skills and proficiency. AI-assisted language learning is an emerging interdisciplinary field that incorporates AI technology into language education theory and practice, aiming to enhance the retention and productivity of language learners by offering personalized, adaptive, interactive, and collaborative learning experiences (Semerikov et al., 2021; Xu et al., 2025). Huang et al. (2023) examined 516 papers published between 2000 and 2019 and found that interest in AI-assisted language instruction was growing (Huang et al., 2023).

Based on the literature review, several significant research gaps remain in the literature. First, little is known about the effects of AI tools on ESL/EFL learners’ emotional variables, such as enjoyment, anxiety, and relevant emotional regulation. Given that learner emotions play a crucial role in language learning, it is necessary to conduct a systematic review focusing on learner emotions in AI-assisted ESL/EFL learning. Second, previous studies mainly focus on how students use AI tools for English vocabulary learning (Xu et al., 2025) and writing development (Lo et al., 2024), which limits our understanding of learner experiences and perspectives with AI tool usage in a comprehensive exploration of the four fundamental English language skills.

To fill these research gaps, this study conducts a systematic review by focusing on the learner emotions in AI-assisted language learning. It aims to clarify the characteristics of the current research into the learner emotions in AI-assisted ESL/EFL learning, emotional variables, and the influence of AI-assisted language learning on L2 learners’ emotional variables.

## 2 Literature review

Several literature reviews have been conducted on the utilization of generative AI tools for English language learning (Alhusaiyan, 2025; Lee et al., 2025; Li et al., 2024; Lo et al., 2024; Wu and Li, 2024; Xu et al., 2025). Lee et al. (2025) analyzed 49 empirical studies published between January 2023 and December 2024, exploring the research design, research focus, and the roles and challenges of GenAI tools in language learning. With language acquisition studies concentrating mostly on AI-assisted writing abilities, they largely examined student perspectives, including their attitude, self-efficacy, and motivation. GenAI performed a variety of functions, such as conversation partner, interaction facilitator, cognitive stimulant, learning tutor, and feedback supplier. The challenges of using AI tools mainly include technological issues like low content quality, information bias, and privacy concerns, and educational challenges, like academic integrity, overreliance, and pedagogical limitations. This study’s suggestions for future research include diverse research designs, more diversified research topics, applying multifaceted roles of GenAI tools with instructional design, and redefining teaching and assessment paradigms in the AI age.

Alhusaiyan (2025) analyzed 15 empirical research articles to investigate the emerging trends and advancements in recently published studies on AI-supported language learning from 2011 to 2022. According to this study, AI technology has the potential to improve language acquisition, especially in areas like learner engagement, scoring accuracy, and writing quality. AI tools were used as intelligent tutoring systems to enhance students’ English writing quality and in automatic writing evaluation to assist EFL students, and motivate them to revise their work. There are still issues with dialogic competence and the need for teacher intervention in educational design. Although AI-supported tools can help people learn languages, more has to be done to encourage language use for collaborative design and communication. This review article highlights the need for more studies on the pedagogical impacts of AI-mediated language learning and the engagement levels of both teachers and students, as well as the significance of investigating the application of AI-mediated language learning in actual classroom environments.

Xu et al. (2025) examined 15 studies to evaluate the effectiveness of AI-supported language learning and analyze factors that influence the effectiveness. All the selected studies were experimental or quasi-experimental in design. AI tools were used to assist English teaching and learning interventions for experimental groups in English listening, speaking, writing, grammar, and vocabulary. As for control groups, conventional instruction and study practices, and conventional materials like printed texts were utilized. The result found that AI technology demonstrated a positive effect on language learning. The type of AI-assisted interaction had no discernible impact on the effectiveness of AI-assisted L2 learning. AI-assisted L2 learning was good at fostering receptive (listening and reading) rather than productive skills (speaking and writing). AI technologies were particularly good at helping learners improve their vocabulary, in comparison to other language skills. Finally, the effectiveness of AI-assisted L2 learning was higher in an in-class setting than an out-of-class one. The implication for instructors is that AI technology should be integrated into classroom instruction, not only for out-of-class study.

Li et al. (2024) analyzed 36 articles to explore the use of ChatGPT in language learning from November 2022 to November 2023. The researchers found that the selected articles from various journals highlight the interdisciplinary nature of this research topic, such as computer science, psychology, linguistics, education, and other social sciences. Empirical research predominates in the published literature, with the majority focusing on higher education and ethical considerations. ChatGPT plays multifaceted roles in facilitating language learning, including content generation, self-directed learning, and teacher workflows. The implication is that it is necessary to understand students’ perceptions of the use of ChatGPT and the relevant impact on their motivation, engagement, and achievement. It is suggested to conduct longitudinal studies to understand the long-term effects on language learning outcomes.

Wu and Li (2024) examined 21 studies published from 2008 to 2023 to investigate the overall effect and moderator analyses of AI tools for EFL learning. All the selected studies were experimental or quasi-experimental using AI tools for English as a foreign language learning, with enough descriptive statistics necessary for effect size calculations. The findings suggest that AI chatbots are useful tools for helping EFL students improve their language proficiency. Additionally, it was discovered that interface designs and intervention durations were important moderators. The implication is that teachers should pay attention to the challenges when using AI chatbots in teaching activities. For example, it may be difficult for non-native speakers to speak with AI chatbots in English, as well as the limited effect of long intervention duration.

Lo et al. (2024) analyzed 70 empirical studies regarding the use of ChatGPT in ESL/EFL education published between November 2022 and June 2024 and provided evidence related to the affordances, such as personalized learning, increased learning opportunities, and teacher support, and potential challenges, such as privacy issue, incorrect feedback, and academic dishonesty of ChatGPT use in ESL/EFL education. Based on this research, most studies have concentrated on how students utilize this AI tool for English writing. Still, very few have looked at how it affects students’ motivation and performance quantitatively. Furthermore, little is known about how ChatGPT affects other language abilities, including speaking, listening, and reading. Accordingly, further research is needed to investigate how ChatGPT can facilitate the teaching and learning of reading and listening skills, as well as a more comprehensive exploration of the four fundamental English language skills.

Based on the above six review articles, two research gaps should be identified. First, they lack a strong focus on learner emotions in AI-assisted ESL/EFL learning, although the six review studies provide insights into AI tool usage for English language education from different angles. Little is known about how the application of AI technology affects learners’ motivation, engagement, performance, and achievement (Li et al., 2024; Lo et al., 2024), as well as other emotion variables. Emotion plays a significant role in language learning; learner and teacher emotions have been considered as the fuel of English learning and teaching (Dewaele and Li, 2020). Accordingly, there is a need to conduct a systematic review of learner emotions in AI-assisted ESL/EFL learning and present an overview of the characteristics of learner emotions in AI-assisted ESL/EFL learning. Second, they lack a comprehensive exploration of the four fundamental English language skills. The majority of previous studies have concentrated on how students utilize AI tools for English vocabulary (Xu et al., 2025) and writing development (Lo et al., 2024). This limits our understanding of learner experiences and perspectives with AI usage in ESL/EFL learning and the relevant English proficiency development.

Driven by the aforementioned research gaps, this article reviews empirical research on learner emotions in AI-assisted ESL/EFL learning with a focus on the characteristics of learner emotions in the utilization of AI technology for ESL/EFL learning, the emotion variables, and the influence of AI-assisted ESL/EFL learning on students’ emotions. The results of this study provide a better understanding of the utilization of AI tools in ESL/EFL learning and their influence on L2 learners’ emotions in recent years. It will be useful for educators and researchers seeking to understand and evaluate AI-assisted ESL/EFL learning, as well as learners interested in using AI tools. The following research questions guide this study.

1. What are the characteristics of the current research into the learner emotions in AI-assisted ESL/EFL learning based on the selected studies?
2. What emotional variables are researched in AI-assisted ESL/EFL learning based on the selected studies?
3. How does AI-assisted ESL/EFL learning influence L2 learners’ emotional variables based on the selected studies?

## 3 Methodology

### 3.1 Research design

**Figure 1:**
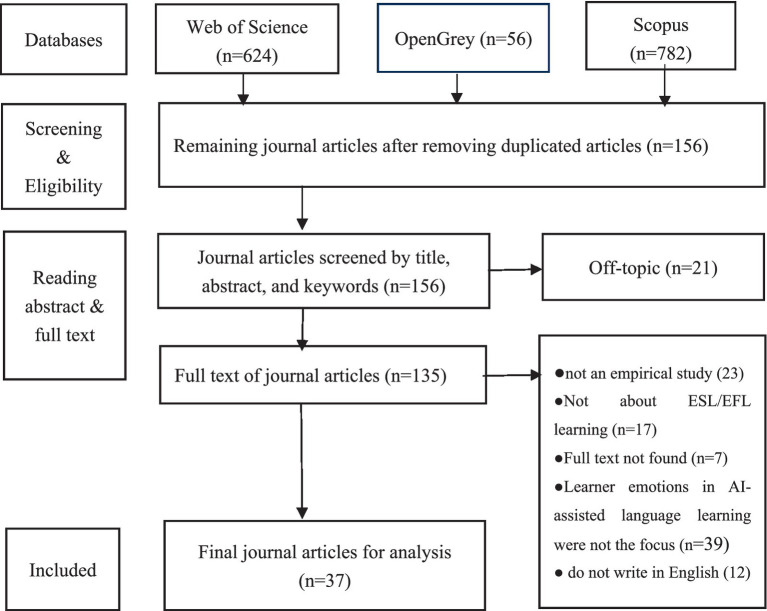
Flowchart process selection.

This study is a systematic review, defined as a comprehensive and unbiased synthesis of the relevant research on specific research questions, involving the identification, selection, synthesis, and appraisal of all qualified evidence (Grant and Booth, 2009). A systematic review aims to gather all the evidence relevant to a research question, focusing on studies that report data rather than concepts or theories. The present systematic review selects journal articles related to the learner emotions in AI-assisted ESL/EFL learning and carries out an analysis of the selected articles based on the three research questions. The Preferred Reporting Items for Systematic Reviews and Meta-Analyses (PRISMA) statement was used to guide this review. PRISMA is an evidence-based collection of guidelines designed to help researchers prepare and report a variety of systematic reviews or meta-analyses and promote thorough and transparent reporting of systematic reviews (Sarkis-Onofre et al., 2021). The whole procedure has been put in the flow diagram based on the PRISMA statement (see Figure 1).

### 3.2 Data collection

To identify potential publications suitable for this study, we searched Scopus, OpenGrey, Web of Science, and Google Scholar for high-quality publications. Regarding the timeframe, there is no time limit on the publication date, so that the researchers can present a comprehensive picture of the learners’ emotions in AI-assisted language learning. The search string used in this review was (emotions OR anxiety OR enjoyment OR engagement OR motivation OR well-being OR positive psychology) AND (artificial intelligence assisted OR AI-assisted OR AI-driven chatbots OR AI chatbots OR ChatGPT OR large language models OR AI-mediated OR AI-enhanced) AND (language learning OR foreign language OR second language OR EFL OR ESL OR English OR L2 learning). To collect the relevant literature and select the high-quality studies, all the publications whose titles, abstracts, or keywords met the search strings were taken into consideration. To avoid the risk of publication bias, grey literature was included in this study and searched in the databases of Scopus, Google Scholar, and OpenGrey.

To refine the results, four inclusion criteria were used: (1) the study is empirical research to meet the overall design of this review; (2) the study is about ESL/EFL learning and writes in English; (3) the study focuses on the learner emotions in AI-assisted language learning; (4) articles published in peer-reviewed journals which had passed the rigorous editorial review and published were selected at first. Then, grey literature, including unpublished theses, preprints, secondary data analysis, book chapters, and conference papers, was examined. Finally, 33 peer-reviewed journal articles and four pieces of gray literature were selected in this study (see Figure 1 and Table 1).

**Table 1:** Quantitative description of the reviewed empirical studies.

Authors	Publication source	AI-based applications	Educational level	Methodology	Data collection method	Experimental	Variables	Findings	Content language learning	Research location
Biju et al. (2024)	Language Testing in Asia	ChatGPT	Higher education	Mixed method	Pre- and posttests, intervention, narrative framework	Yes	Foreign language anxiety (FLA), attitudes, motivation, and writing skills	AI-assisted assessment greatly raised learners’ motivation, improved attitudes about language acquisition, and lowered FLA. The pretest writing abilities across groups showed no appreciable variation. Even though the difference was not statistically significant on the posttest, the experimental group exceeded the control group. The results of this study imply that AI-assisted assessments can generate a helpful learning environment, lower anxiety, improve attitudes, and increase motivation, thereby delivering useful information.	Writing	Bangladesh
Chen et al. (2024)	Acta Psychologica		Higher education	Quantitative	Questionnaires	No	AI self-efficacy, AI-related anxiety, and students' overall attitude toward AI	AI self-efficacy could negatively affect AI anxiety, and positively influence both learners’ attitude toward AI and their actual use of AI tools. Besides, AI anxiety negatively predicted the actual use of AI. Moreover, AI self-efficacy was a positive predictor of AI use through reducing AI anxiety, enhancing attitude toward AI, or a combination of both.	College English	China
Derakhshan (2025)	Learning and Motivation	Generative AI	Higher education	Qualitative	Semi-structured interview	No	Emotional engagement, goal orientation, and motivational climate	Effective use of GAI technologies in L2 education can affect students’ emotional, cognitive, and behavioral states. While GAI-mediated instruction is new to many educators, it can engage and tap into learners’ emotionality and goal orientation. The nature of such technologies involves one’s emotions and goal setting because they change almost everything in the class, from relationships to actions.		Iran
Elov et al. (2025)	Language Testing in Asia	LinguaTest platform	Higher education	Quantitative	Pre- and posttests, intervention, questionnaire	Yes	Shyness, foreign language anxiety, autonomy, and enjoyment	Applying Intelligent Computer-Assisted Language Assessment in performing oral tests may moderate students’ shyness, foreign language anxiety, autonomy, and enjoyment in language assessment.	Speaking	Uzbekistan
Elsayed et al. (2024)	Language Testing in Asia		Higher education	Quasi-experimental	Pre- and posttests, intervention, questionnaires	Yes	Student demotivation, anxiety, L2 learning experiences, and academic success	Teacher support in AI-assisted exams can substantially benefit L2 learners. Additionally, the findings indicate that AI-assisted exams can considerably improve learning outcomes when paired with effective teacher involvement, highlighting implications for various stakeholders.	General English course	Ethiopia
Fan and Zhang (2024)	Heliyon		Higher education	Quantitative	Questionnaires	No	AI literacy, learner attitudes, foreign language enjoyment, continuance intention	AI literacy, learner attitudes, and FLE significantly predict the CI. Moreover, AI literacy indirectly influences CI by mediating learner attitudes and FLE. Finally, FLE mediates the relationship between learner attitudes and CI. The study verifies a sequential mediation model, providing new insights into EFL learners’ cognitive and affective traits and extending current models on the continuance intention in AIassisted EFL learning.		China
Guo and Wang (2025)	European Journal of Education	ChatGPT, Wenxin Yiyuan, Bodoudou	Higher education	Mixed method	Questionnaire, semi-structured interview	Yes	Academic engagement, emotional experience, emotional engagement	Integrating AI into EFL instruction has a positive effect on students' cognitive, emotional and social engagement. Moreover, the learners' emotional experiences were found to be abundant and dynamic, exerting influence on their academic engagement.	English literature	China
Lee et al. (2023)	RELC Journal	AI-based content generator	Secondary school	Quantitative	Survey, intervention	Yes	Foreign language enjoyment and interest	It was found that the condition based on the artificial intelligence-based content generator-based activity was more effective in terms of enhancing the target variables, and that the group which engaged in the artificial intelligence-based content generator-based activity was largely in favor of artificial intelligence-based content generator technology.	Reading	China
Khasawneh et al. (2025)	Language Testing in Asia		Higher education	Quasi-experimental	Pretests and posttests, intervention, questionnaire	Yes	Portfolio assessment in AI-assisted environments, academic emotion regulation, mindfulness, and attitudes toward language learning	The potential of AI-assisted portfolio assessment in enhancing AER and mindfulness while fostering positive attitudes toward language learning. By comparing AI-integrated and traditional approaches, this study provides valuable insights into the role of AI in promoting effective and engaging learning experiences in EFL contexts.	Portfolio assessment in AI-assisted environments	Ethiopia
Kohnke and Moorhouse (2025)	Computers in Human Behavior		Higher education	Qualitative	Semi-structured interview	No	Emotional engagement, motivation and well-being	The findings revealed that GenAI generally enhances students’ motivation, reduces anxiety and stress, and fosters an emotionally supportive learning environment. However, challenges related to cultural context and technical issues were also identified. The study highlights the pivotal role of instructors in shaping students’ experiences with GenAI and underscores the need for ongoing support and professional development. It also demonstrates the importance of cultural sensitivity, technological infrastructure and balance.	English proficiency	China
Liu et al. (2025)	European Journal of Education	ChatGPT or similar tools	Higher education	Mixed method	Questionnaire, semi-structured interview	No	Ideal L2 Self, Foreign language enjoyment, engagement, confidence, and willing to practice	Students who participated in AI-IDLE more often reported a clearer ideal L2 self and greater FLE, but those with a greater ideal L2 self did not report more FLE. EFL learners can acquire a sense of FLE and vivid ideal L2 selves as they agentively negotiate the affordances of generative AI for informal language learning purposes, the sense of FLE and motivational force may shift across contexts to shape their continued investment in AI-IDLE practices.	AI informal digital English learning	China
Luo and Zou (2024)	Learning and Motivation	ChatGPT	Higher education	Quantitative	Questionnaires	No	Enjoyment and basic psychological needs	The result indicated a positive impact of enjoyment on basic psychological needs factors. In addition, the results revealed no gender differences among enjoyment and basic psychological needs factors and no significant difference in the relation between enjoyment and basic psychological needs after controlling the impact of gender.		China
Ma and Chen (2024)	BMC Psychology		Higher education	Quasi-experimental	Pre-and posttests, intervention	Yes	Student engagement and academic procrastination	The results indicate that the experimental group, exposed to AI-empowered applications, demonstrated significantly higher levels of affective, cognitive, and behavioural engagement than the control group. The study underscores the potential of AI-empowered applications to enhance learner engagement and mitigate academic procrastination.		China
Pawlak et al. (2025)	Computer Assisted Language Learning	CorpusMate and ChatGPT	Higher education	Quantitative	Questionnaires	Yes	Task enjoyment, task anxiety, and task boredom	Task enjoyment increased, while boredom and anxiety decreased over time in AI-assisted L2 writing. At the within-individual level, task anxiety showed the highest stability, followed by task enjoyment, with task boredom being the least persistent. Findings also indicated that task emotions exerted significant reciprocal influences on each other, with stronger impacts from task enjoyment. At the between-individual level, learners with higher levels of task enjoyment and boredom did not necessarily carry these emotions over into future sessions, whereas learners with higher task anxiety tended to maintain this emotional state over time. Additionally, higher levels of task enjoyment and lower levels of task anxiety and boredom predicted better L2 writing outcomes, with the persistence of these emotions playing a crucial role.	Writing	Iran
Rezai et al. (2024)	European Journal of Education	ChatGPT	Higher education	Quantitative	Questionnaire	No	Well-being and emotion regulation	The outcomes of structural equation modelling revealed a strong mediation effect of emotion regulation in the relationship between using ChatGPT and well-being. Additionally, significant positive correlations were found between using ChatGPT and both well-being and emotion regulation. Besides, a significant positive relationship was established between emotion regulation and well-being among the EFL learners.	English as a Foreign Language	Iran
Shen and Tao (2025)	International Journal of TESOL Studies	Quillbot and DeepL	higher education	Quantitative	Questionnaires	No	Writing anxiety	This study revealed metacognitive strategies including planning, monitoring and evaluating, and AIbased writing self-efficacy have a negative impact on EFL learners’ writing anxiety in AI-assisted writing contexts. In addition, AI-based writing self-efficacy fully mediates the relationship between planning strategies and learners’ writing anxiety. It also partially mediates the relationships between monitoring strategies and writing anxiety, as well as between evaluating strategies and writing anxiety within AIassisted writing contexts.	English writing	China
Shi (2025)	Journal of Educational Computing Research		Higher education	Mixed method	Pre- and posttest, intervention, semi-structured interview, survey	Yes	Emotional self-regulation and linguistic proficiency	The results indicate significant improvements in language retention and emotional regulation for learners using AI tools. Qualitative feedback from interviews and surveys corroborates these findings, underscoring the positive impact of AI on educational experiences. This research highlights the potential of AI to deepen emotional engagement and tailor learning experiences, recommending the incorporation of AI technologies into language learning programs to boost linguistic competencies and enrich educational outcomes.	English linguistic proficiency	China
Wang et al. (2024)	International Review of Research in Open and Distributed Learning	Poe	Higher education	Quasi-experimental	Pretest and posttest measures, questionnaire	Yes	Writing anxiety, writing complexity, fluency, and accuracy	The group that received AI-generated feedback performed better than the group that received teacher feedback or no AI support. Additionally, learners in the AI-generated feedback group experienced a more significant reduction in writing anxiety than their peers. These results highlight the remarkable impact of AI-generated CF on improving writing outcomes and alleviating anxiety in undergraduate language learners.	Writing	China
Wang Yu (2025)	British Educational Research Journal		Higher education	Mixed method	Pre- and posttest, intervention, open-ended questionnaire	Yes	Anxiety, enjoyment, and overall English proficiency.	The quantitative results indicated that the integration of the AI‐powered tool significantly reduced the learners’ anxiety and significantly enhanced their learning enjoyment. Furthermore, the outcomes showed a significant promotion in the overall English proficiency of the experimental group compared with the control group. Qualitative findings complemented these results, detailing participants’ positive perceptions of how AI support beneficially influenced their emotional states and language development.	Language proficiency	China
Wang et al. (2025b)	Learning and Motivation	ChatGPT, Grammarly	Higher education	Quasi-experimental	Pre- and posttest, intervention, questionnaire	Yes	Emotional regulation, motivation, psychological well-being, and academic writing proficiency	Both AI-powered tools significantly improved emotion regulation and psychological well-being while positively impacting the students' motivation and the quality of their academic writing. These results lend support to the potential of AI-powered tools to foster holistic student development within the specific context of Chinese higher education and offer valuable insights for stakeholder seeking to integrate AI effectively into similar learning environments.	Writing	China
Wang et al. (2025a)	European Journal of Education	Doubao, EAP talk	Higher education	Quantitative	Questionnaire, pre- and posttest	Yes	Various emotions and willingness to communicate	EFL learners with higher communicative confidence and greater foreign language learning boredom tend to perceive GenAI chatbots as less useful for developing speaking skills. While GenAI successfully aided them in improving their speaking skills through both theme-based and free dialogues, learners who are more willing to engage in face-to-face interactions with peers and teachers may not find chatbots as productive or engaging as human counterparts.	Speaking	China
Wu and Liu (2025)	Discover Computing		Higher education	Quantitative	Questionnaires	No	Enjoyment, boredom, and curiosity, cognitive processing, and the ideal L2 self	Gender did not affect the constructs under study, but GenAI competence positively influenced speaking performance. Enjoyment had a direct effect on cognitive processing, which in turn enhanced speaking performance, though it did not influence the ideal L2 self. Curiosity positively influenced the ideal L2 self and speaking performance, but had no effect on cognitive processing. Boredom, however, did not affect either cognitive processing or the ideal L2 self.	Speaking	China
Yu and Tao (2025)	British Educational Research Journal		Higher education	Quasi‐experimental	Pre- and posttests, intervention	Yes	Academic emotion regulation, self‐esteem, second language (L2) learning experiences and growth mindsets	EG demonstrated significantly higher levels of academic emotion regulation, self‐esteem, L2 learning experiences and growth mindsets compared to the CG on the post‐tests. These findings underline the potential of AI‐integrated learning environments to positively shape learners' emotional, psychological and cognitive outcomes.		China
Xin and Derakhshan (2025)	European Journal of Education		Higher education	Qualitative	Semi-structured interview, narrative framework	No	Positive emotions, negative emotions, emotion consideration and regulatory strategies.	Chinese EFL students had mostly experienced positive emotions of ‘motivation’, ‘excitement’, ‘engagement’ and ‘confidence’. On the negative side, they reported experiencing ‘frustration’, ‘anxiety’, and ‘stress’ more frequently in their classes. Furthermore, the study indicated that the participants had used six strategies, namely ‘seeking help from others’, ‘shifting attention’, ‘cognitive change’, ‘persistent practice’, ‘staying positive’ and ‘suppression’ to regulate their AI-induced emotions.	AI-induced emotions and regulation strategies	China
Xiao et al. (2024)	International Review of Research in Open and Distributed Learning	ChatGPT; Duolingo	Higher education	Quantitative	questionnaire; test	Yes	Self-esteem, cognitive-emotion regulation, academic enjoyment, and language success	AI-supported online classes can play in improving students’ psychological well-being as well as their engagement in their academic work. Enhanced Self-Esteem increased EFL learners’ engagement. Self-Esteem guided the cognitive-emotion regulation of EFL learners. The interactive speaking activities, aided by AI, led to improvements in EFL learners’ (a) speaking and listening skills, (b) critical thinking abilities, (c) affective engagement, and (d) language success.	Speaking and listening	China
Xu and Thien (2025)	Journal of Applied Research in Higher Education		Higher education	Quantitative	Questionnaires	No	Perceived enjoyment, intrinsic motivation, students' intention to use ChatGPT for English learning	Findings showed that effort expectancy, performance expectancy, social influence and perceived enjoyment were positively related to Chinese undergraduate EFL learners' intention to use ChatGPT for English learning. Perceived enjoyment mediated the relationships between effort expectancy, performance expectancy, social influence and intention to use ChatGPT for English learning respectively.		China
Xu et al. (2024)	Heliyon	ChatGPT	Higher education	Quantitative	Questionnaire	No	Foreign language self-efficacy and foreign language enjoyment,	The use of ChatGPT has a significant positive impact on both the foreign language self-efficacy and foreign language enjoyment of Chinese students studying abroad. Furthermore, foreign language enjoyment also positively influences foreign language self-efficacy. In addition, foreign language enjoyment serves as a mediator between the use of ChatGPT and foreign language self-efficacy.		China
Yang et al. (2025)	British Educational Research Journal		Higher education	Mixed method	Pre-posttest, intervention, semi-structured interview	Yes	Positive emotions, technostress and psychological well-being	Learners in the AI with teacher support group demonstrated significantly higher levels of positive emotions, lower technostress, and enhanced psychological well-being compared to both the AI without teacher support group and the CG. Qualitative analysis further highlighted three key themes for the AI with teacher support group: teacher-mediated emotional security and positive affect; teacher buffering of AI-induced technostress; and teacher–AI synergy enhancing psychological well-being. In contrast, the AI without teacher support group emphasised autonomy-driven motivation, technological resilience, and a pronounced craving for guidance. The findings highlight the importance of integrating teacher support with AI tools to foster a more balanced and effective learning environment.		Malaysia
Zhang S. (2025)	British Educational Research Journal		Higher education	Quantitative	Questionnaires	No	Emotion regulation strategies, grit, self‐compassion, L2 learning experiences and academic demotivation	Emotion regulation strategies were positively associated with L2 learning experiences and negatively associated with academic demotivation. Similarly, grit tendencies demonstrated positive correlations with L2 learning experiences and negative correlations with academic demotivation. Self‐compassion demonstrated similar patterns, with positive relationships to L2 learning experiences and negative associations with academic demotivation.		China
Zhang and Liu (2025)	Innovation in Language Learning and Teaching		Higher education	Quantitative	Questionnaires	No	Students’ burnout, emotional dysregulation, and emotional well-being	EFL students experience fluctuations in their emotional states throughout the course, with burnout and emotional dysregulation typically increasing due to workload, stress, and academic challenges. Some students, however, benefit from improvements in emotional well-being through positive feedback and social interactions. The integration of AI-driven platforms was found to influence students’ initial emotional states, with personalized learning experiences helping to alleviate burnout but potentially leading to dysregulation for some students. Additionally, AI tools that provided continuous support and adaptive feedback contributed to a slower increase in burnout and a more stable emotional state over time. However, the effectiveness of AI-driven platforms in predicting these changes depends on the tools’ design and students’ engagement with the technology.	English proficiency	China
Zhang et al. (2024)	System	an AI-speaking assistant, Lora	Higher education	Quasi-experimental	Pre- and post survey, intervention	Yes	Foreign language enjoyment (FLE), foreign language anxiety (FLA), and willingness to communicate (WTC) in English	Results unveiled significant enhancements in WTC and FLE, accompanied by a noteworthy reduction in FLA among the AI-engaged EG. Conversely, the CG exhibited no significant changes in those variables. These findings underscore the efficacy of AI-speaking assistants in amplifying EFL students’ FLE and WTC while mitigating FLA.	Speaking	China
Zhang et al. (2025)	Behavioral Sciences		Higher education	Quantitative	Questionnaire	No	Willingness to communicate, self-efficacy, foreign language classroom anxiety, AI literacy	AI literacy improves self-efficacy in AI learning and diminishes classroom anxiety, both of which are significant mediators in the relationship between AI literacy and willingness to communicate. The study highlights the imperative of integrating AI literacy into EFL instruction to enhance learners’ expressive confidence and mitigate fear.	Speaking	China
Zong and Yang (2025)	European Journal of Education		Higher education	Quantitative	Questionnaire	No	Engagement, emotional well-being, learning outcomes	AI-enhanced social–emotional learning significantly boosts student engagement and emotional well-being. By providing tailored learning experiences based on students' emotional and cognitive needs, AI systems facilitate better emotional regulation, increased focus and improved academic performance.		China
Gray literature										
Deng et al. (2024)	Proceedings of the 2024 International Conference on Innovation in Artificial Intelligence		Higher education	Mixed-method	Questionnaire, open-ended questions	No	Willingness to communicate	AI's role in pre-talk preparation significantly contributed to the success of students' interactions, emphasizing the value of AI chatbots in the absence of authentic communication opportunities. The study concludes that strategically integrating AI chatbot aligns with the active filter hypothesis, reducing anxiety and promoting positive WTC, thus enhancing language learning experiences.	Speaking	China
Ma (2024)	Unpublished Master Thesis, University of Washington		Higher education	Qualitative	In-depth interview	No	Language learning difficulties, identity negotiation, and code-switching complexity	Findings suggest that AI tools provide adjunctive support by providing personalized explanations, improving learning efficiency, and reducing language-related anxiety.		the US
Ali et al. (2025)	Book chapter		Higher education	Quantitative	Survey	No	Language testing anxiety	Higher anxiety levels were associated with increased AiLTs usage. Notably, familiarity with AiLTs did not show a clear link to test anxiety. These results emphasize the importance of considering test anxiety when assessing AiLTs usage.		Malaysia
Hayashi and Sato (2024)	Conference paper	ChatGPT	Higher education	Mixed-method	Test and questionnaire	Yes	English language proficiency, Foreign language anxiety	The preliminary findings demonstrated a significant reduction in L2 anxiety and an improvement in English interaction skills. These results suggest that generative AI has the potential to transform language learning and offer innovative approaches to contemporary teaching methods.		Japan

The initial search using the keyword string yielded 624 articles in the Web of Science database, 56 articles in OpenGrey, and 782 articles in the Scopus database. The researchers screened the title, abstract, and keywords to determine whether the article should be included in this study based on the predefined inclusion criteria. We filtered publications that were not within the scope of this study by the following exclusion criteria: (1) lacking empirical data; (2) not about EFL/ESL learning or in English; (3) not focusing on the learner emotions in AI-assisted language learning. Finally, 37 articles were included in the present study. Figure 1 shows the selection procedure of publications for this systematic review.

### 3.3 Data analysis

The technology-based learning review (TLR) paradigm proposed by Hsu et al. (2012) was used to code the selected publications. The factors of “Research purposes,” “Application domains,” and “sample groups” should be considered to examine the trends in technology-based learning. Additionally, the elements that link the three dimensions, such as “adopted technologies/learning environments,” “research issues,” and “research methods,” should also be included (Hsu et al., 2012). In order to examine the target studies, we also consulted the coding schemes developed by other previous studies (Chang and Hwang, 2019; Zhang and Hasim, 2023).

Eleven categories were used in this study to address the three research questions: authors, publication source, AI-based applications, educational level, methodology, data collection method, experimental or not, emotion variables, findings, content language learning, and research location.

## 4 Findings

### 4.1 Research characteristics of the learner emotions in AI-assisted language learning

This study synthesized data through a systematic review, following the PRISMA statement, to ensure transparency and comprehensiveness (Sarkis-Onofre et al., 2021). Data collection involved Scopus, Web of Science, OpenGrey, and Google Scholar, with a focus on empirical studies in English and an examination of learner emotions in AI-assisted ESL/EFL contexts. This resulted in 33 peer-reviewed journal articles and 4 pieces of grey literature, totaling 37 studies. Data coding used the technology-based learning review (TLR) paradigm (Hsu et al., 2012), with 11 categories. Table 1 provides an overview of the selected empirical studies related to the learner emotions in AI-assisted ESL/EFL learning.

According to Table 1, all the selected journal articles were published in the years 2023, 2024, and 2025, which indicates that learner emotion in AI-assisted language learning is a relatively new field of research, partly because the release of ChatGPT in November 2022 and its application in language learning attracted increasingly more academic attention from then on. Researchers of the selected studies used a variety of AI-based applications, such as ChatGPT, Weixin Yiyuan, Bodoudou, Xunfei Xinghuo, Doubao, Deepseek, and EAP talk, which indicate that AI tools play a significant role in ESL/EFL learning and instruction.

**Figure 2:**
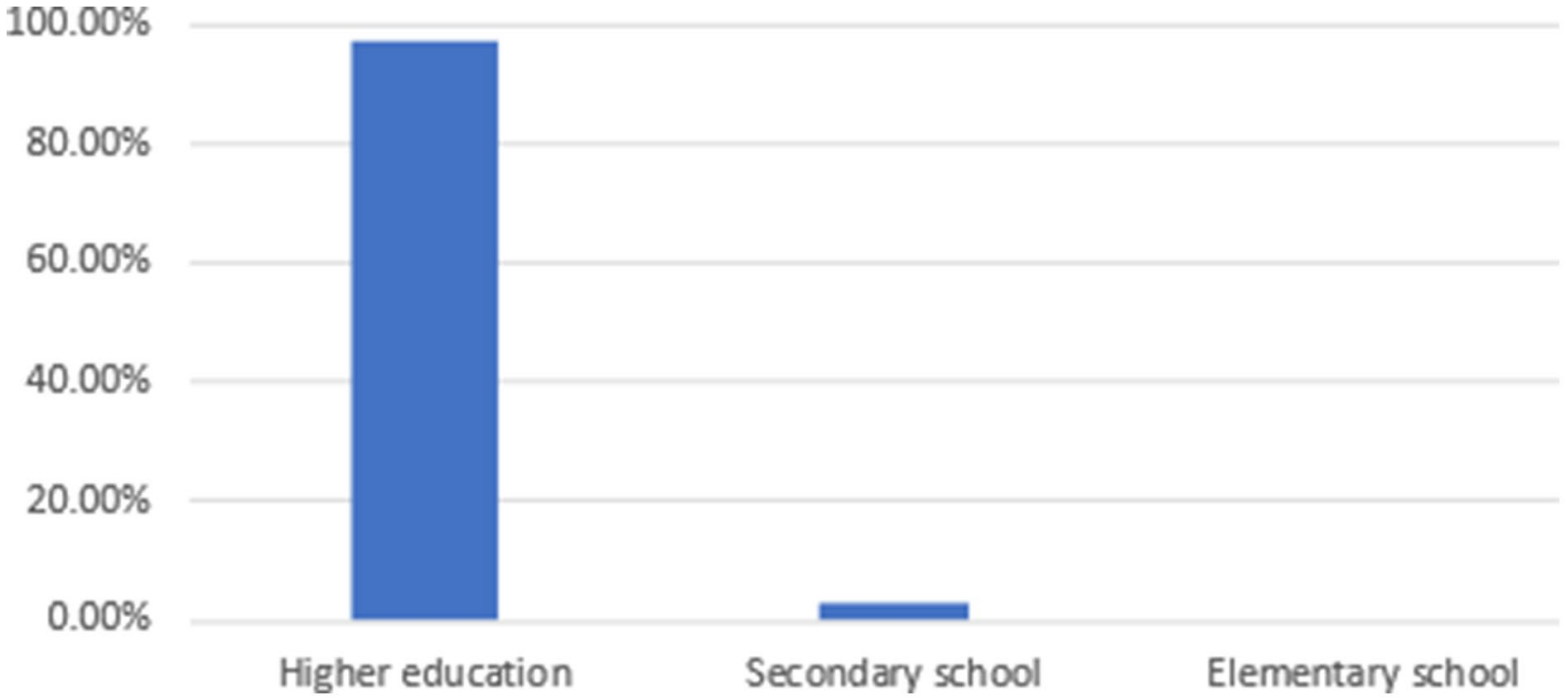
The distribution of educational level.

**Figure 3:**
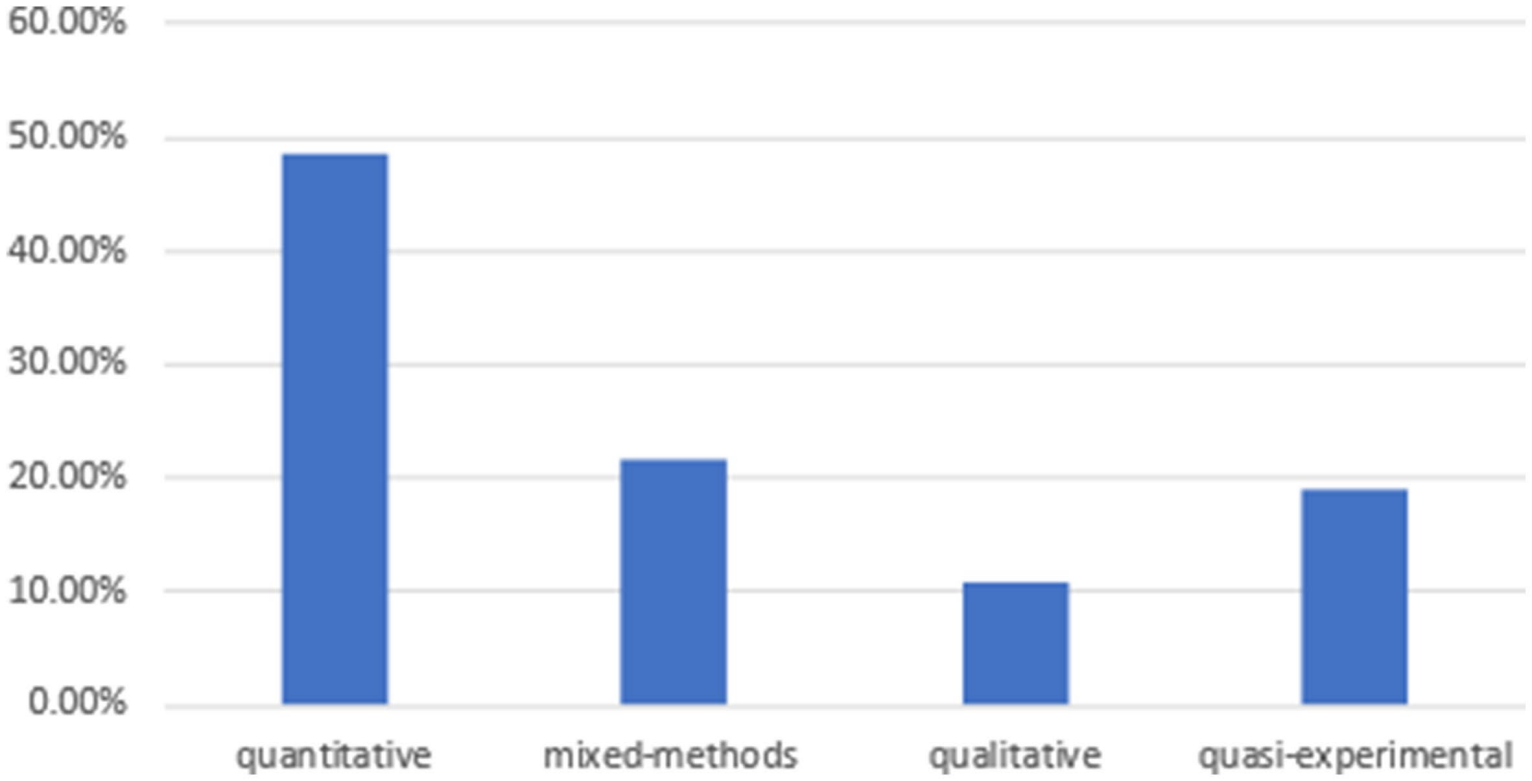
The distribution of research methods.

As for the distribution of educational level (see Figure 2), higher education accounts for 97%, followed by secondary school (3%). No elementary school participants were mentioned in the selected studies. Eighteen out of 37 selected articles (Nearly half) are experimental. Multiple research methods were used in the selected studies (see Figure 3), with quantitative (48.6%), mixed-methods (21.7%), qualitative (10.8%), and quasi-experimental (18.9%). Various data collection methods are utilized in the selected studies: pre- and posttests, interventions, questionnaires, narrative framework, interviews, and so forth. The content of language learning involves English speaking, listening, reading, writing, English for academic purposes, vocabulary, literature, assessment, grammar, and so forth. Some of the selected studies did not mention the specific content of language learning. These selected empirical studies were conducted in various ESL/EFL countries, including Afghanistan, Bangladesh, China, Ethiopia, Indonesia, Iran, Iraq, Malaysia, Oman, Saudi Arabia, Spain, and Uzbekistan.

### 4.2 Emotional variables researched in the reviewed studies

Table 2 presents a variety of emotional variables in AI-assisted language learning in the selected studies. The most frequently researched variables are learner anxiety and enjoyment, followed by learner boredom, interest, and shyness. Besides, learners’ emotion regulation is also an important topic studied in the selected studies.

**Table 2:** The emotion variables researched in the publications reviewed.

Positive emotion variables	Frequency (N)	Negative emotion variables	Frequency (N)	Emotion regulation	Frequency (N)
Enjoyment	13	Anxiety	15	Emotion regulation	9
Interest	1	Boredom	3	Emotional engagement	5
		Shyness	1		

Negative emotions like learner anxiety, boredom, and shyness were researched in the selected studies. Wang et al. (2025a) and Wang et al. (2025b) found that learners with greater foreign language boredom and higher communicative confidence tend to consider AI chatbots as less useful for developing English speaking skills, although AI tools might be useful in improving their speaking skills. Chen et al. (2024) considered that students’ AI anxiety could negatively predict their actual use of AI tools. A student who feels anxious about using AI tools might avoid using AI-powered study apps, writing tools, or research assistants, even if those tools could be helpful. This point suggests that addressing AI anxiety could potentially encourage greater adoption and use of AI tools among students.

Positive emotions, like enjoyment and interest, were also researched. Learner enjoyment can have a positive impact on the basic psychological needs factor (Luo and Zou, 2024). Fan and Zhang (2024) posited that foreign language enjoyment, as well as AI literacy and learner attitudes, significantly predicted students’ usage of AI tools and their continuance intention. Furthermore, foreign language enjoyment can mediate the relationship between learner attitudes and their continuance intention in AI-assisted language learning. However, a wider range of emotional variables may coexist among learners. Therefore, some of the selected studies have combined several emotional variables (Chen et al., 2024; Derakhshan, 2025; Elsayed et al., 2024; Pawlak et al., 2025; Yang et al., 2025; Wang, 2024; Xin and Derakhshan, 2025; Zhang et al., 2024). For example, Zhang et al. (2024) conducted a quasi-experimental study to examine the influence of Lora, an AI-speaking assistant, on Chinese EFL students’ foreign language anxiety (FLA), enjoyment (FLE), and willingness to communicate (WTC) in English learning. This study consisted of the experimental (*n* = 65) and control groups (*n* = 66). And the intervention lasted for 6 weeks. Based on the results of the pre- and post-intervention survey, the AI-engaged experimental group showed a notable decrease in foreign language anxiety, with notable improvements in willingness to communicate and foreign language enjoyment. On the other hand, the control group showed no significant changes in those variables. These results highlight the usefulness of AI-speaking assistants in amplifying EFL students’ FLE and WTC while reducing FLA.

Learners’ emotion regulation is another crucial research topic (Yu and Tao, 2025; Zhang, 2025; Zhou and Hou, 2024; Zong and Yang, 2025). Yu and Tao (2025) found that students who utilized AI tools in English learning demonstrated significantly higher levels of academic emotion regulation. AI-empowered applications significantly improve learners’ emotional, cognitive, and behavioral engagement (Ma and Chen, 2024; Shi, 2025; Wang et al., 2025a; Wang et al., 2025b), as well as mitigating academic procrastination (Ma and Chen, 2024). Similarly, Rezai et al. (2024) found that there was a positive correlation between EFL learners’ emotion regulation and their use of ChatGPT. ChatGPT has the potential to foster the EFL learners’ emotional regulation because it may provide a non-judgmental platform for them to process and express their feelings. Therefore, successfully integrating AI tools into EFL learning might promote students’ holistic development within the context of higher education (Wang et al., 2025a; Wang et al., 2025b).

### 4.3 The influence of AI-assisted language learning on learner emotions

As shown in Table 1, all the selected studies confirmed the positive influence of AI-assisted language learning on learners’ emotions, as the effective application of AI tools in L2 education can affect students’ emotional, cognitive, and behavioral states (Derakhshan, 2025), thus positively influence students’ well-being and emotion regulation (Rezai et al., 2024; Yu and Tao, 2025) to foster learning outcomes. The most frequently researched emotional variables are learner anxiety and enjoyment. Additionally, AI-driven assessment is a rapidly growing research topic.

Much scholarly attention is paid to foreign language anxiety (Fan and Zhang, 2024; Shen and Tao, 2025; Wang, 2024). In AI-assisted writing environments, EFL learners’ writing anxiety is negatively impacted by AI-based writing self-efficacy. The relationship between learners’ writing anxiety and planning strategies is fully mediated by AI-based writing self-efficacy. Additionally, AI-based writing self-efficacy partially mediates the relationship between writing anxiety and the use of monitoring strategies (Shen and Tao, 2025). Similarly, both Wang et al. (2024), Wang et al. (2025a), and Wang et al. (2025b) found that students in the AI-generated feedback group experienced a more significant decrease in writing anxiety and better learning outcomes than their peers.

Foreign language enjoyment also receives much academic attention (Luo and Zou, 2024; Xin and Derakhshan, 2025; Xu et al., 2024; Zhang et al., 2024). Cognitive processing was directly impacted by enjoyment, which improved students’ learning outcomes (Wu and Li, 2024). It was found that AI-assisted L2 writing can increase task enjoyment while decreasing boredom and anxiety, and predict better L2 writing outcomes (Pawlak et al., 2025). Using AI technology has a positive impact on students’ foreign language enjoyment and willingness to communicate (Zhang et al., 2024), and foreign language self-efficacy (Xu et al., 2024). Foreign language enjoyment can mediate the relationship between foreign language self-efficacy and the use of AI technology, as well as positively influence foreign language self-efficacy (Xu et al., 2024). Students who participated in AI-assisted informal language learning often reported greater foreign language enjoyment and a clearer ideal L2 self, which encourages their continued investment in AI-IDLE practices (Liu et al., 2025).

AI-driven assessment is another hot research topic. AI-assisted portfolio assessment can enhance students’ academic emotional regulation while fostering positive attitudes and promoting students’ mindfulness toward language learning (Khasawneh et al., 2025). AI-assisted assessment significantly enhanced participants’ vocabulary knowledge and emotional resilience (Khan et al., 2024). AI-driven assessment has significantly improved learners’ motivation and attitudes toward language acquisition, while also reducing foreign language anxiety, thereby creating a helpful learning environment and providing useful information (Biju et al., 2024; Kohnke and Moorhouse, 2025). Using AI Language Assessment in oral English tests may moderate students’ shyness, anxiety, autonomy, and enjoyment in language assessment (Elov et al., 2025). These results highlight the significance of integrating advanced technologies into educational frameworks to foster students’ cognitive and emotional development (Khan et al., 2024; Khasawneh et al., 2024; Zhang, 2025; Zhang and Liu, 2025).

However, using AI technology in ESL/EFL learning is not a perfect approach. The challenges of AI-assisted language learning involve concerns about reduced human interaction (Du and Alm, 2024), cultural context, and technical issues (Kohnke and Moorhouse, 2025). Students emphasize the significance of human-teacher empathy and interactivity while acknowledging the benefits of AI technology (Du and Alm, 2024). It is crucial to pay attention to the cultural sensitivity, technological infrastructure, and balance (Kohnke and Moorhouse, 2025).

## 5 Discussion

This review article aims to provide readers with the research characteristics of learner emotions in AI-assisted ESL/EFL learning, the relevant emotional variables, and the effects of AI-assisted ESL/EFL learning on learner emotions in the selected studies. Thirty-seven relevant studies were selected based on the inclusion criteria.

Regarding RQ1, it was found that research on learner emotions has proliferated. The relevant empirical studies were conducted in more than 10 non-English-speaking countries. This point is consistent with the finding of Huang et al. (2023) that learners’ interest in AI-assisted language learning is increasing. The integration of AI technology in foreign language learning is increasingly popular.

Unlike some previous studies (e.g., Lee et al., 2025; Lo et al., 2024), which claimed that AI tools were mainly used to cultivate English writing skills, the findings of this study were that AI tools could be widely used in the cultivation of English listening, speaking, reading, writing, vocabulary, grammar, English for academic purpose, English literature, assessment, and informal digital English learning. This indicates the feasibility and practicality of applying AI tools for actual ESL/EFL classrooms.

The findings of this study agree with some previous studies (e.g., Lee et al., 2025; Li et al., 2024) that higher education is the dominant setting in research on AI-assisted ESL/EFL learning, partly because university students have higher linguistic competence and cognitive maturity, enabling them to engage with sophisticated AI technologies that might be too complex for younger learners. Another likely reason is easier data collection among university students (Lee et al., 2025). This limits our understanding of the influence of AI-assisted ESL/EFL learning on primary and secondary school students, as well as the effectiveness of AI technology in the K-12 learning environment. Compared to higher education, K-12 students need different pedagogical strategies and developmental considerations (Lee et al., 2023). Findings from higher education may be different from those in K-12 contexts due to the differences in digital literacy, learner agency, and institutional infrastructure between K-12 and higher education (Lee and Jeon, 2024). Accordingly, future studies are suggested to address the impacts of AI-assisted ESL/EFL learning in K-12 education for a comprehensive understanding across all educational levels.

Regarding RQ2, the reviewed studies highlight several emotional variables in AI-assisted ESL/EFL learning, with notable variations in research focus and methodology. This finding aligns with Dewaele and Li's (2020) study that research on learner emotions heralds an interest in both positive and negative emotions in the current situation. Based on Dewaele and Li (2020), research on learner emotions in second language acquisition can be divided into three phases. The first phase could be called the Emotion Avoidance Phase (between the early 1960s and the mid-1980s), because emotions were considered an irrational factor in language learning. The second phase is called the Anxiety-Prevailing Phase (between the mid-1980s and the early 2010s), many relevant studies focused on the negative emotion of anxiety. The third phase is called the Positive and Negative Emotions Phase (from the early 2010s to now). Influenced by positive psychology, both positive and negative emotions are researched in language teaching and learning. The selected studies are from the years 2023, 2024, and 2025, which witness the diverse emotions in L2 learning, as well as the complex and dynamic interactions between positive and negative emotions in the AI-assisted ESL/EFL learning context. For example, Zhang et al. (2024) showed that AI intervention simultaneously improved Willingness to Communicate and Foreign Language Enjoyment while reducing Foreign Language Anxiety, suggesting that AI tools can address multiple emotional dimensions synergistically.

This study adds that learner anxiety (N = 15) and enjoyment (N = 13) are the most frequently investigated variables in AI-assisted ESL/EFL learning based on Table 2, reflecting their central role in learners’ affective experiences. The frequency data in Table 2 further reveal disparities in research focus. Variables like emotion regulation (*N* = 9) are moderately explored, while constructs such as boredom (*N* = 3), interest (*N* = 1), and shyness (*N* = 1) remain under-explored. This research gap suggests a need to broaden the affective scope of AI-assisted language learning. Emotional variables, like boredom, interest, hope, and feeling scared, are worthy of further research.

Regarding RQ3, the selected studies unanimously confirm the positive impact of AI-assisted ESL/EFL learning on learners’ emotional states, demonstrating that AI technology can systematically shape affective, cognitive, and behavioral dimensions in second language education. This point aligns with previous studies (e.g., Dizon, 2020; Wu and Li, 2024), which suggest that AI-assisted language learning can increase learners’ positive emotions and decrease their negative emotions. As mentioned in Table 1, AI-driven tools utilize personalized assessment and adaptive feedback to serve as learning partners rather than instruments, addressing individual needs in vocabulary, grammar, pronunciation, and other aspects (Aldosari, 2024; Derakhshan, 2025).

The benefits of AI-assisted ESL/EFL learning on learner emotions can be analyzed from four aspects. Firstly, it is found that learner engagement emerges as a central construct, with AI interventions consistently enhancing cognitive, emotional, and social engagement (Guo and Wang, 2025; Hao and Abu Bakar, 2025). For example, AI-powered chatbots and writing tools have been shown to mitigate academic procrastination (Ma and Chen, 2024) and improve writing ability (Hastomo et al., 2025), while gamified AI interfaces boost task enjoyment (Pawlak et al., 2025). This aligns with Huang’s (2024) assertion that engagement acts as a catalyst for language learning efficiency, highlighting AI’s role in sustaining learners’ active participation.

Secondly, AI-assisted ESL/EFL learning helps trigger foreign language enjoyment in the learning process (Lee et al., 2023; Xiao et al., 2024; Xu and Thien, 2025). Enjoyment mediates the relationship between self-efficacy and AI use, with AI-assisted tasks consistently increasing learners’ pleasure and willingness to communicate (Zhang et al., 2024; Xu et al., 2024). For instance, AI-based writing platforms reduce boredom and anxiety while predicting better writing outcomes (Pawlak et al., 2025), and informal AI learning environments foster a clearer ideal L2 self (Liu et al., 2025). These findings support the affective filter hypothesis, suggesting that AI technology alleviates psychological barriers to enhance cognitive processing (Wu and Li, 2024).

Thirdly, AI technology demonstrates significant anxiolytic effects, particularly in writing contexts. Shen and Tao (2025) found that AI-based writing self-efficacy fully mediates the relationship between writing anxiety and planning strategies, while Wang et al. (2024) and Wang Yu (2025) reported greater anxiety reduction in AI-feedback groups compared to traditional instruction. This may occur because AI provides structured, error-focused feedback without the social evaluation threats inherent in human interaction, allowing learners to take risks and build confidence.

Finally, AI-powered assessment tools not only improve language skills but also cultivate resilience and positive mindsets (Abdellatif et al., 2024; Khan et al., 2024). Portfolio assessment systems, for example, enhance emotional regulation and mindfulness (Khasawneh et al., 2025), while oral English AI tests moderate shyness and anxiety (Elov et al., 2025). The non-threatening nature of AI assessment may encourage learners to view errors as learning opportunities rather than failures, promoting growth mindsets.

## 6 Conclusion, implications, and limitations

This systematic review investigates learner emotions in AI-assisted ESL/EFL learning through reviewing 37 selected empirical studies. The findings reveal that research in this field has surged since 2023, driven by the proliferation of AI tools (e.g., ChatGPT) and their integration into language education. Both positive and negative emotions were researched. Key emotional variables, including enjoyment and anxiety, dominate the literature, with AI technologies consistently demonstrating positive effects on learners’ affective states. For instance, AI-powered tools enhance cognitive, emotional, and social engagement and mitigate learning anxiety. Additionally, this study highlights that AI-assisted learning impacts not only English writing but also listening, speaking, reading, and vocabulary, underscoring its versatility in language skill development. However, the field remains nascent, with most studies focusing on higher education settings and overlooking the relevant emotional complexities in K-12 contexts.

### 6.1 Implications

There are several implications for future research. First, policymakers should leverage the review’s findings to support equitable and effective integration of AI technology in ESL/EFL education. It is necessary to invest in AI infrastructure and accessibility, particularly in resource-constrained regions, to ensure all learners can benefit from AI technology, regardless of socioeconomic status. Second, researchers are suggested to address key gaps to deepen the understanding of learner emotions in AI-assisted ESL/EFL learning. For instance, expand the scope of emotional variables, such as interest and boredom, to understand their roles in AI-assisted language learning. Then, diversify educational contexts. It is necessary to investigate the impact of AI technology on emotions in K-12 settings to bridge the current higher education bias. In addition, most studies employ quantitative designs with short intervention periods (e.g., 6–8 weeks), leaving long-term emotional impacts unexamined. Therefore, it is suggested to adopt longitudinal designs to explore AI tools’ sustained emotional effects on ESL/EFL learning. Third, educators should leverage AI tools to design emotionally supportive environments, such as using AI chatbots for low-stakes speaking practice to reduce anxiety or adaptive feedback systems to boost motivation.

### 6.2 Limitations

This study has several limitations. First, all reviewed studies were published between 2023 and 2025, reflecting the recency of AI tools like ChatGPT in education. This limits insights into long-term trends or the stability of emotional impacts over time. Second, with 97% of studies focusing on higher education, findings may not apply to K-12 learners, who have distinct emotional and cognitive needs, such as greater dependence on teacher guidance or lower digital literacy. Third, there is limited diversity in emotional variables. This review emphasizes anxiety and enjoyment, but under-researched emotions (e.g., hope, shame) may play significant roles in AI-assisted learning. This narrow focus overlooks the complexity of learners’ affective experiences. Finally, methodological imbalance exists. Quantitative studies (48.6%) dominate, with fewer qualitative (10.8%) or mixed-method (21.7%) designs. This limits understanding of the subjective emotional experiences that quantitative data may miss.

## Data Availability

The original contributions presented in the study are included in the article/supplementary material, further inquiries can be directed to the corresponding author/s.
